# Backcasting COVID-19: a physics-informed estimate for early case incidence

**DOI:** 10.1098/rsos.220329

**Published:** 2022-12-14

**Authors:** G. A. Kevrekidis, Z. Rapti, Y. Drossinos, P. G. Kevrekidis, M. A. Barmann, Q. Y. Chen, J. Cuevas-Maraver

**Affiliations:** ^1^ Department of Applied Mathematics and Statistics, Johns Hopkins University, Baltimore, MD 21218, USA; ^2^ Department of Mathematics and Carl R. Woese Institute for Genomic Biology, University of Illinois at Urbana-Champaign, Urbana, IL 61820, USA; ^3^ European Commission, Joint Research Centre, I-21027 Ispra (VA), Italy; ^4^ Department of Mathematics and Statistics, University of Massachusetts Amherst, Amherst, MA 01003, USA; ^5^ Grupo de Física No Lineal, Departamento de Física Aplicada I, Universidad de Sevilla. Escuela Politécnica Superior, C/ Virgen de África, 7, 41012 Sevilla, Spain; ^6^ Instituto de Matemáticas de la Universidad de Sevilla (IMUS). Edificio Celestino Mutis. Avda. Reina Mercedes s/n, 41012 Sevilla, Spain

**Keywords:** COVID-19, embedding theorems, epidemics, time series, Gaussian process

## Abstract

It is widely accepted that the number of reported cases during the first stages of the COVID-19 pandemic severely underestimates the number of actual cases. We leverage delay embedding theorems of Whitney and Takens and use Gaussian process regression to estimate the number of cases during the first 2020 wave based on the second wave of the epidemic in several European countries, South Korea and Brazil. We assume that the second wave was more accurately monitored, even though we acknowledge that behavioural changes occurred during the pandemic and region- (or country-) specific monitoring protocols evolved. We then construct a manifold diffeomorphic to that of the implied original dynamical system, using fatalities or hospitalizations only. Finally, we restrict the diffeomorphism to the reported cases coordinate of the dynamical system. Our main finding is that in the European countries studied, the actual cases are under-reported by as much as 50%. On the other hand, in South Korea—which had a proactive mitigation approach—a far smaller discrepancy between the actual and reported cases is predicted, with an approximately 18% predicted underestimation. We believe that our backcasting framework is applicable to other epidemic outbreaks where (due to limited or poor quality data) there is uncertainty around the actual cases.

## 1. Introduction

During the early stages of COVID-19 (and in fact of any emerging disease), even estimating the basic reproduction number *R*_0_ was a challenge [1–4] due to lack of information and the absence or poor quality of data. *R*_0_ is defined as the number of secondary infections an infectious individual can generate in a population of susceptible individuals. It is quite important for epidemiologists and public health officials to have an accurate estimate of its magnitude.

The clinical characteristics of the disease—latency period, period of infectiousness and incubation period—were not known, so differing estimates of *R*_0_ existed (ranging from 1.4 to 6.49) [5] that largely depended on the models that were used to estimate them and the corresponding assumptions made. At the same time, limited testing capacity obscured the true size of the epidemic and the actual growth rate.

Varying estimates of symptomatic and asymptomatic cases also exist [6–8]. For example, and of relevance to the time series we consider in this work, [9] estimated that the level of under-reporting of infected individuals in the Italian region of Lombardy at the beginning of the first wave was severe. They used an epidemiological model with compartments for both asymptomatically and symptomatically infected individuals to estimate that on 8 March 2020 the calculated cumulative number of asymptomatically infected cases was of the order of 10–15 times the confirmed cumulative number of infected cases (see also the estimates of under-reporting for different countries in the work of [10]). The importance of asymptomatics and that of social distancing was further explored in [11], via an extended version of the model developed in [9] whereby two additional epidemiological compartments were introduced (hospitalized and quarantined individuals). These inadequacies, in turn, caused serious handicaps in mounting an appropriate response by public health authorities. While our understanding of the relevant models and both their benefits and weaknesses has greatly progressed [12–14], there is still significant room for improvement of our understanding of both the first and subsequent waves of the epidemic.

As the epidemic progresses and more data become available—daily new cases, number of tests performed, daily new hospital admissions and daily new deaths—these too should be evaluated critically. It is now widely known, and generally accepted, that COVID-19 reported data are neither reliable nor complete and that the best practice is to base mathematical models on hospitalization and fatality time series data [13]. Reported-case count time series are unreliable due to the limited number of tests available initially, and the large number of cases that are asymptomatic [7] and/or go unreported [15]. A recent systematic review and data meta-analysis concluded that 35.1% (95% confidence interval (CI): 30.7—39.9%) were asymptomatic infections [8].

It is then natural that various methods and studies exist attempting to reconstruct the true number of case counts and fatalities, or better, to provide an improved or more reliable estimate. In [16], a backcasting (BC) approach based on fatalities and a gamma distribution of the time from infection to death was used to study the epidemic in 15 countries. It was estimated that the number of infected people is 6.2 (95% CI: 4.3–10.9) times higher than reported. This echoes a study of the epidemic in the USA [17], where probabilistic bias analysis was used to approximate the true case counts. They found that 89% of infections were probably undocumented. Indeed, the US Centers for Disease Control indicated an under-reporting of infections by a factor of 2 to 13 times in [18], illustrating the gravity and relevance of the issue. In Brazil, it was estimated [19] that the actual case counts are three times and the deaths are twice as many as those reported. A study of the epidemic dynamics around the globe [20] using a Bayesian Gaussian process model found large disparities in the degree of under-reporting among countries. Cumulative infection data from several European countries were studied using the so-called capture–recapture (CRC) methods [21], and it was found that the ratio of calculated total over observed (i.e. reported) cases was around 2.3. In [22], we chose to neglect infection data and instead focused on fatalities, for a study of the epidemic in Mexico. Fitting the model to the infection time series produced unreliable predictions of future deaths due to the under-reporting of infections.

While fatalities are believed to be more reliable than case counts [22], care should still be taken when using them as a benchmark due to different ways of measuring and reporting the data among countries [23]. For instance, excess deaths, rather than reported deaths, during the first wave in the northern part of Italy were embedded in a differences-in-differences regression model. One of the findings of the study was that deaths may be under-reported by as much as 60% [24]. The reason for the discrepancy is that only hospital deaths are included in the official reports.

The excess mortality in the first months of the epidemic was estimated to be 28% higher than the reported COVID-19 fatalities. Another pertinent point is that delays in death counts may be as long as a year in some cases [25]. In fact, [26] found that in three countries (United States, India and Brazil) with similar federal structure, the offset between the case and death time series was country specific and depended on the COVID-19 wave (first, second and third). This limits our ability to produce accurate, real-time forecasts if recent data are not reliable; but if the majority of the data is eventually counted and correctly revised, then this situation lends well to a retrospective analysis after, say, a year.

In the present study, we develop and apply a method that can be used to extrapolate from the second (and generally later) wave, the fatalities and the COVID-19 infections. We assume that during the second wave, both reported infections and reported fatalities are more accurate and are used together with the fatalities during the first wave (also assumed to be accurate) to backcast the reported infections during the first wave. A word of caution on the accuracy of the reported number of cases during the second wave is in order. Monitoring protocols throughout the pandemic were neither uniform in time nor within a country. For example, in Italy, during the first dramatic wave, regions followed different protocols: the Veneto region had a policy of extensive testing, whereas Lombardy, Piedmont and initially Emilia Romagna [27] tested only symptomatic individuals. As a result, Veneto had a lower increase in mortality. Similarly, a study of the time-lag between cases and deaths in the USA from approximately the start of the epidemic to May 2021, when the CDC changed its reporting policy, found that the time-lag followed an ‘up-down-up’ pattern [28]. This pattern was attributed to delays in testing and limited treatment (first wave), improved treatment and non-pharmaceutical interventions (second wave) and worsening conditions during the third wave as measures were relaxed. Hence, a combination of individual behaviour and reporting policy might render reported cases less accurate than expected as the pandemic progressed. Similar arguments, for example treatment improvements or infection of different age groups, also apply for the accuracy of reported fatalities, as mentioned at the end of §2.2.1. Finally, as previously mentioned, the number of asymptomatics and their contribution to the total number of cases is difficult to quantify. In the rest of this work, we shall refer to the reported cases during the second wave as accurate, bearing in mind, however, these limitations and reservations as a potential topic for further study. Our methodology can be applied to countries whose COVID-19 time series are characterized by a first peak, succeeded by a period of very low daily incidence during the summer months (i.e. between the first and the second wave), followed by a second peak in the autumn. By using the second wave time series as training sets for our algorithm, we carry out this programme for a substantial number of countries, principally from Europe, and also from Asia and the Americas, obtaining a consistent under-reporting of the relevant diagnostics.

Our presentation is structured as follows. First we provide the mathematical background for our analysis in §2.1. Subsequently, we present the relevant data and their structure in §2.2, comment on uncertainty in diagnosed cases in §2.3, and present a schematic of our methodology in §2.4. In §3, we present our numerical findings, and §4 summarizes critically our findings and provides directions for the future work. The electronic supplementary material contains further information on our methodology, describes model parameters and addresses issues concerning data uncertainty and hyperparameter tuning.

## 2. Method

### 2.1. Theory: delay embedding

Our operating assumption is that we are given the time series of *reported cases* (*C*) and *reported fatalities (deaths)* (*D*) per day in a geographical region. More specifically, we denote

2.1 $$D={\left\{d_{t}\right\}}_{t\geq 0}^{T}$$

and

2.2 $$C={\left\{c_{t}\right\}}_{t\geq 0}^{T},$$

where *d*_*t*_ and *c*_*t*_ denote the fatalities and officially diagnosed cases on day *t*, for *t* = 0, 1, 2, …, *T*. We further assume that *D* is *accurate* for all *t*, while *C* is only accurate after time *t* ≥ T* and *inaccurate* for *t* < T*, where T* is a time point chosen between the first and second waves, chosen as explained in the following section.

Suppose that *d*_*t*_ and *c*_*t*_ are discrete observations of two corresponding quantities *D*(*t*) and *C*(*t*) that satisfy an *n*-dimensional system of coupled deterministic ordinary differential equations whose dynamics feature a unique attractor. Then, the state of the system at any given time can be represented by an *n*-dimensional vector and the overall behaviour of the system can be represented on a manifold $M\subset R^{n}$.

According to the delay embedding theorems of Whitney and Takens [29–32], we are able to use only *d*_*t*_ to construct a manifold $M_{D}\subset R^{k}$ that is diffeomorphic to M. Such a manifold is guaranteed to exist whenever the embedding dimension *k* satisfies *k* ≥ 2*n* + 1: that is, there exists a differentiable invertible transformation (diffeomorphism) $f\;:\;M_{D}\to M$. Since *c*_*t*_ is known after time T*, we can use that part of the dataset to *learn* (using regression) the restriction of *f* to the ‘reported-cases’ coordinate

2.3 $$\hat{f}∼f{|}_{C}\;:\;M_{D}\to C(t),\;t\geq T^{*}.$$

We proceed to evaluate $\hat{\;f}$ on the points of the reconstructed manifold $M_{D}$ observed for *t* ≤ T* to estimate *c*_*t*_ during the early stages of the pandemic.

### 2.2. Data

In electronic supplementary material, appendix A we summarize the sources of our data and we comment on their quality. In addition, we address issues of data uncertainty, and we comment on the reliability of the time series used and on the embedding and Gaussian process parameters.

#### 2.2.1. Structure and T*

The framework proposed in §2.1 is very general in that it only requires two time series of the observed quantities (*C*, *D*) and some knowledge of the time T*.

T* is the time after which *C* can be considered *accurate*, and it may be hard to define. The naive definition of accurate and inaccurate is *true* and *false*, respectively, meaning that we take all information after time T* at face value. It is arguably more natural to think of the *accuracy* of the considered data as a continuously varying quantity as opposed to a Boolean variable. In principle, it may be possible to incorporate additional statistics such as (but not limited to) the amount of testing [33] and vaccination rates [34] to quantify the uncertainty of the data. However, this process would complicate our analysis since the availability, quality and adequacy of additional markers is not certain, and their consideration would result in a more complex but less applicable model.

**Figure 1.**
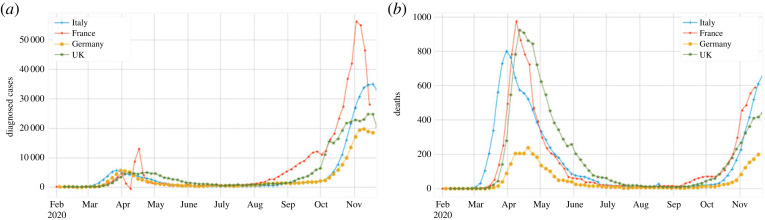
Seasonal structure of the (*a*) case and (*b*) death time series for the UK and the three most populous countries in the European Union (EU; Germany, France, Italy) during the first months of 2020. (The UK officially exited the EU after 31 January 2020.) Few diagnosed cases and deaths were observed during the summer months.

Fortunately, there is structure in many time series at the country and local levels that can be exploited. Several countries in Europe experienced a first peak in the observed cases and fatalities, as shown in figure 1*a*,*b*, respectively. This was followed by a decline during the summer months, before the second peak in the autumn. Thus, a heuristic definition of T* can be described as the time when mortality falls close to zero for a long period, which coincides, in many countries, with the summer months of 2020. The leads to an *almost Boolean* T* occurring some time within this period.

We note that our BC results are not sensitive to such a T*, as long as its choice is reasonable, namely, that it falls within the period of low mortality and not close to the second peak (see §3.1 for the effect of the choice of T* on BC results for Italy). A compromise in the characterization of *accuracy* can be reached by assuming that the data after T* are not necessarily true, but can be trusted enough to make *an estimate* of the true number of infections after T*, which is further explored in §2.3.

An additional complication arises due to varying mortality rates as the epidemic progressed. For instance, a study for Sweden [35] spanning data from March to September 2020 found that mortality in hospitalized patients decreased as the pandemic progressed. This may be attributed to improvements in treatment protocols, the availability of new treatments (e.g. remdesivir, monoclonal antibodies), differences in the virulence of circulating variants [36] or differing age distributions of those infected in the first versus second wave [37]. The inability to address these issues fully is why our results are qualified as approximate estimates of the true case incidence.

### 2.3. Uncertainty in diagnosed cases

We are interested in building an uncertainty estimate around the diagnosed cases we use during our calibration period, which will, in turn, correspond to uncertainty around the backcasted result. We turn to the literature to obtain a 'hidden-case' estimator that preserves some of the integrity of our current data.

The CRC method presented in [21] estimates that if the diagnosed cases at time *t* are *c*_*t*_, then the true number of cases at time *t* is a random variable ${\hat{c}}_{t}$, where [21,38]

2.4 $$E[{\hat{c}}_{t}]=c_{t}+\frac{{\left(c_{t}\right)}^{2}}{c_{t-1}-d_{t}}$$

and

2.5 $$V[{\hat{c}}_{t}]=\frac{{\left(c_{t}\right)}^{4}}{{\left(1+c_{t-1}-d_{t}\right)}^{3}}+\frac{4{\left(c_{t}\right)}^{3}}{{\left(1+c_{t-1}-d_{t}\right)}^{2}}+\frac{{\left(c_{t}\right)}^{2}}{1+c_{t-1}-d_{t}}.$$

Thus, the case time series after T* can be adjusted (accompanied by a 95% normal-based CI) using case and fatality observations after T*. This estimator only depends on our available time series information and does not take into account any external prior knowledge (such as serial interval distribution). This is desirable because we believe that post-T* diagnosed case counts are of much higher fidelity than early ones, and we would like our estimates to be based on them since they are true observables of the system behaviour we are trying to capture.

Our results show that this estimate cannot be used reliably in this problem framework *without* BC, since ${\hat{c}}_{t}$ still depends on the validity of *c*_*t*_, which is not close to the true number of cases before T*. We choose to present the main part of our numerical results using CRC to quantify uncertainty, aside from figures 5*b* and 6 in §3.1, where we also present the incorporated Gaussian process (GP) uncertainty estimates. The uncertainty estimation method, however, is arbitrary, and one can choose it independently as part of the BC algorithm.

### 2.4. The backcasting algorithm

**Figure 2.**
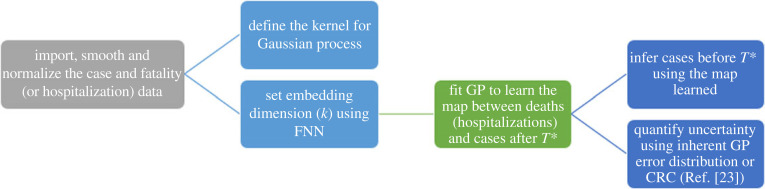
Flowchart of the proposed BC algorithm. FNN, false nearest neighbours (method); GP: Gaussian process (regression); CRC: capture--recapture (method).

Figure 2 summarizes the algorithm we used to backcast the number of cases, given two time series. We chose the time series of reported number of cases and number of fatalities. First, the embedding dimension *k* was determined via the false nearest neighbours (FNNs) [39] (or alternatively average false neighbours [40]) algorithm as implemented in the Python nonlinear time series module nolitsa^1^ [41,42]. Then, we used Gaussian process (GP) regression (see electronic supplementary material, appendix B) to obtain ${\hat{f}}_{\mathrm{GP}}$, a process that requires learning the case time series after time T* and then using it to backcast the number of cases before T*. We used the sklearn^2^ Python library [43] to perform the Gaussian process regression. Finally, we quantified uncertainty estimates using the CRC method of [21]. We note in passing that further work on the use of GP for learning dynamical system behaviour in the same manner can be found in [44].

Importantly, each step of the proposed algorithm is independent. One may use GP if other reliable uncertainty estimates are not available, but may also use another regression method to learn the estimated function. Similarly, one may also use a separate uncertainty method specific to the country or dataset the algorithm is applied to. This is impractical when working with multiple countries, but it will lead to more accurate results when optimizing these choices for a specific dataset. The theory behind the validity of the algorithm is valid regardless of the method applied.

In the electronic supplementary material, we provide further information on the definition and use of the embedding parameters (the delay time *τ* and the embedding dimension *k*, appendix A) along with Gaussian process parameters and the corresponding kernels used, appendix B.

We conclude the methodological section by noting that the reconstructed (backcasted) cases time series may be used to provide improved estimates of the disease reproduction numbers. The basic and effective reproduction numbers (*R*_0_ and *R*_*e*_, respectively) are interpretable parameters that measure the number of further infections caused by a single-case incidence in a population of susceptible individuals at a given time *t*. They are natural measures of the spread of an epidemic as they provide a quantitative measure of its potential spread. As such, they are important in public health policy decisions during an outbreak. They can appear explicitly in compartmental models, whether purely statistical or parametric (in the case of a differential equation model), in which case their relationship with the observed time series is model dependent. Russo *et al.* [9] performed such an analysis for the reproduction numbers for the Italian dataset using a compartmental epidemiological model (hence, an ordinary-differential-equations model). Perhaps also relevant to this work is the R-package ‘R0’^3^ [45] that provides a general toolbox for the estimation of both reproduction numbers based on incidence data.

## 3. Numerical results

### 3.1. Italy: a case study

**Figure 3.**
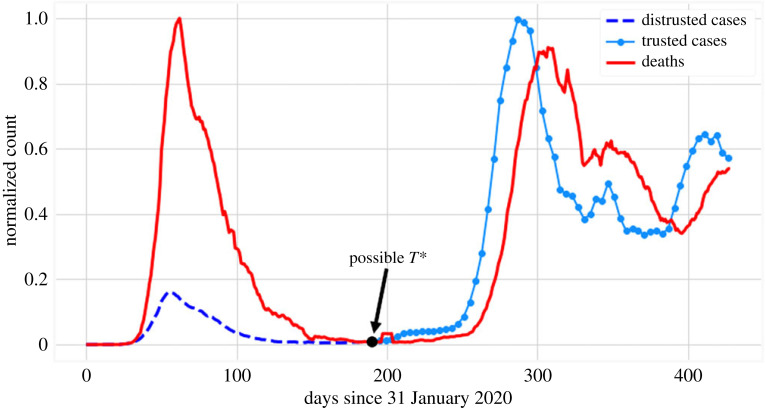
Normalized diagnosed cases and deaths in Italy. We divide each series by its maximum to be able to compare both quantities in one graph (i.e. *c*_*t*_/max_*t*_{*c*_*t*_}, *d*_*t*_/max_*t*_{*d*_*t*_}, where $\max_{t}\{c_{t}\}=35\;073,\;\max_{t}\{d_{t}\}=814$, respectively).

We analyse the time series of a single country as a prototypical case example: Italy. In figure 3, we present the normalized time series for Italy from the beginning of the pandemic. While the fatalities in the two *waves* beginning at approximately *t* = 30 and *t* = 250, respectively, are of the same magnitude, we note that the diagnosed cases during the first wave are much lower than the second wave would suggest. In this case, a nominal T* is identified, but it could be specified to be at any point where the incidence of both cases and fatalities is very low (i.e. during the summer months).

**Figure 4.**
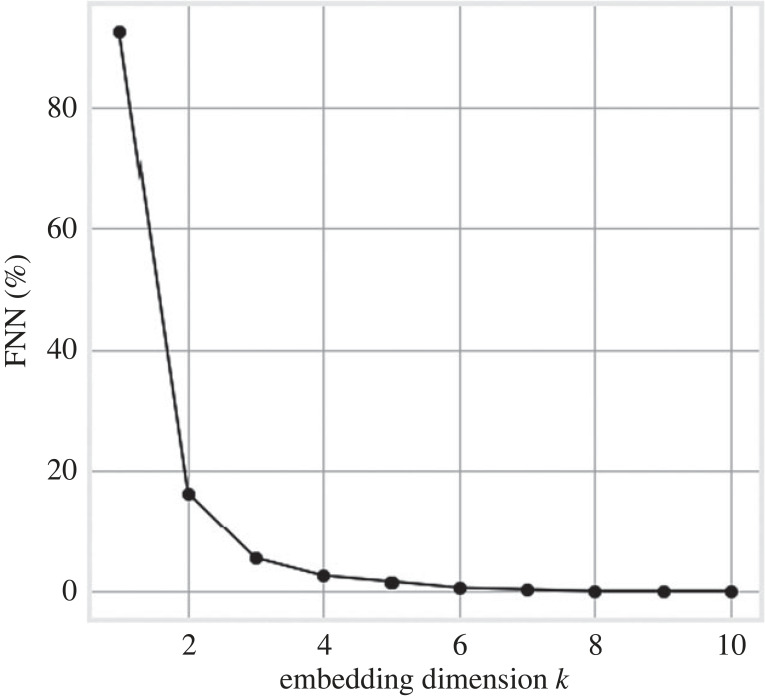
Number (in %) of false nearest neighbours for the death time series of Italy under the Chebyshev metric (see figure 3). The algorithm is implemented through the Python module nolitsa.

Because the disparity between the number of reported cases of the two waves suggested by figure 3 is not trustworthy, and since the data satisfy the structure presented in §2.2.1, we claim that this is a good candidate time series to backcast. By using the FNN algorithm, we estimate the embedding dimension for the fatality time series to be *k* = 10; see figure 4. Here, the percentage of false neighbours drops to 0 at that dimension. We use the Chebyshev metric (*L*_∞_) due to its lower sensitivity to additive noise. Note that this computation is dataset specific and is repeated for each dataset we use (table 1). In addition, we arbitrarily but consistently, as discussed, pick T* = 160 occurring during the period of low case and death incidence.

**Table 1.** Estimates of the embedding dimension *k* of the death time series for each country.

country	embedding dimension (estimate, FNN)	start date
Brazil	5	26 February 2020
France	8	24 January 2020
Germany	9	27 January 2020
Italy	10	31 January 2020
South Korea	12	21 January 2020
Spain	5	1 February 2020
Sweden	7	1 February 2020
UK	6	31 January 2020

Given the embedding dimension, we construct $M_{D}$ such that $d_{t}=\{d_{t-9},\ldots ,d_{t}\}$ $\in M_{D}$, proceeding to fit our GP estimator

3.1 $${\hat{\;f}}_{\mathrm{GP}}(d_{t})=c_{t},\;\forall t\geq 160,$$

and then backcast to the initial cases ${\hat{c}}_{t}$

3.2 $${\hat{c}}_{t}={\hat{\;f}}_{\mathrm{GP}}(d_{t}),\;\forall t\in [10,160].$$

**Figure 5.**
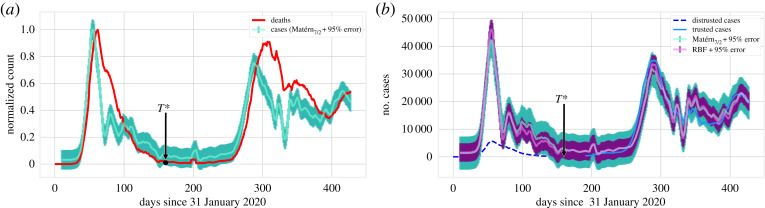
(*a*) Normalized backcasted cases (using the Matérn kernel with *ν* = 7/2 (electronic supplementary material, appendix B )) and deaths in Italy, in analogy to figure 3. (*b*) BC result for Italy with a time window of 10 days. Results for two kernels (radial basis function (RBF) and Matérn (*ν* = 7/2)) are presented for the GP regression.

**Figure 6.**
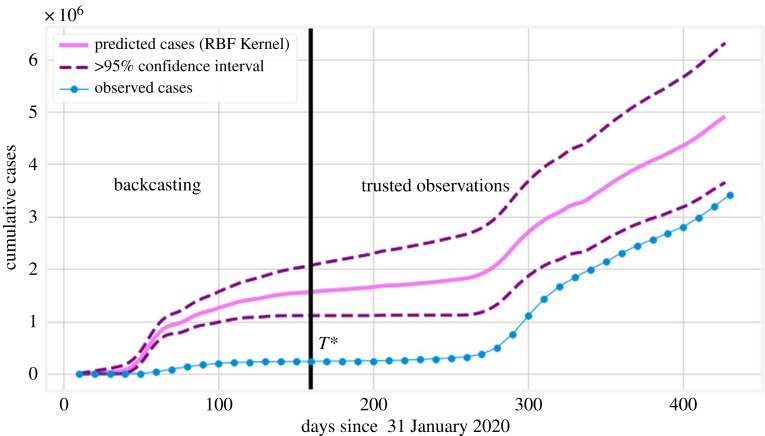
Cumulative BC result for diagnosed cases in Italy (with a time window of 10 days) corresponding to the daily results of figure 5*b*. This figure only presents one of the results (RBF) for the sake of clarity. After time T* = 160, the predicted cases (pink, solid line) are almost an exact translation of the observed cases (blue, solid line, filled circles) since the model has been trained at those points.

We obtain the result as shown in figures 5 and 6. A remarkable feature of figure 5*b* is that at the peak of the first wave, the number of reported daily cases was found to be less than 10 000, while any selection of the kernel in our BC scenarios suggests that there were approximately 40 000 cases at that time. Even by adjusting for the trusted observations in figure 6, we still find a substantial disparity between the number of cumulative cases observed and the ones predicted by our BC analysis. The uncertainty interval of this and similar figures is computed based on the uncertainty of the corresponding daily cases and is greater than 95% based on the sub-additivity of standard deviation. As we consider more data, the disparity (as a ratio) between observed and estimated cases will necessarily decrease because the result is cumulative and the increments agree after T*. Comparing cumulative cases at T* gives a more accurate picture of the beginning stages of the pandemic, while comparing at a later time can be used to understand the long-term effect of that early (unreported) spike in cases, see e.g. the results presented in tables 2 and 3.

**Table 2.** Summary of the figure 10 BC predictions for the cumulative number of diagnosed cases at time T* (9 July 2020) and at the end of the considered period (4 April 2021). Two different predictors were used: death or hospitalization times series, both coupled with CRC uncertainty estimates.

backcasted cumulative number of cases (>95% CI)					
country	reported number of cases ×10^6^	backcasting based on death time series		backcasting based on hospitalizations	
		number ×10^6^	backcasted to reported	number ×10^6^	backcasted to reported
9 July 2020—T*					
Italy	0.24	1.68 (1.61, 1.75)	7.01 (6.73, 7.29)	1.46 (1.40, 1.52)	6.08 (5.83, 6.34)
France	0.20	1.89 (1.82, 1.96)	9.27 (8.93, 9.61)	2.17 (2.09, 2.25)	10.66 (10.27, 11.04)
Sweden	0.08	0.61 (0.56, 0.66)	7.97 (7.35, 8.58)	0.48 (0.44, 0.53)	6.33 (5.80, 6.87)
UK	0.28	2.20 (2.13, 2.28)	7.73 (7.46, 8.01)	1.57 (1.50, 1.64)	5.51 (5.28, 5.74)
4 April 2021—final date					
Italy	3.59	5.13 (5.93, 5.33)	1.43 (1.37, 1.48)	4.89 (4.70, 5.08)	1.36 (1.31, 1.42)
France	4.70	6.54 (6.31, 6.77)	1.39 (1.34, 1.44)	6.84 (6.60, 7.08)	1.45 (1.40, 1.51)
Sweden	0.80	1.35 (1.25, 1.46)	1.69 (1.56, 1.82)	1.22 (1.12, 1.32)	1.53 (1.40, 1.65)
UK	4.36	6.40 (6.17, 6.62)	1.47 (1.42, 1.52)	5.75 (5.54, 5.96)	1.32 (1.27, 1.37)

**Table 3.** Summary of the figure 11 BC predictions for the cumulative number of diagnosed cases at time T* (9 July 2020) and at the end of the considered period (4 April 2021). The death times series coupled with CRC uncertainty estimates was used as a predictor.

backcasted cumulative number of cases (>95% CI)			
country	reported number of cases ×10^6^	backcasting based on death time series	
		number ×10^6^	backcasted to reported
9 July 2020—T*			
Germany	0.19	1.03 (0.98, 1.08)	5.30 (5.03, 5.58)
South Korea	0.01	0.03 (0.02, 0.4)	2.64 (1.83, 3.46)
Spain	0.29	2.12 (2.03, 2.21)	7.38 (7.06, 7.70)
4 April 2021—final date			
Germany	2.84	3.79 (3.62, 3.96)	1.33 (1.27, 1.40)
South Korea	0.10	0.13 (0.09, 0.16)	1.22 (0.91, 1.53)
Spain	3.28	5.17 (4.95, 5.38)	1.57 (1.51, 1.64)

**Figure 7.**
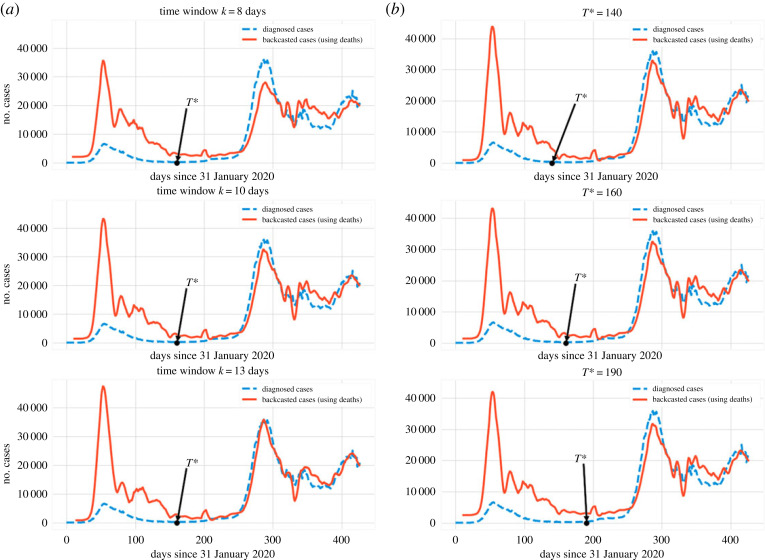
(*a*) The qualitative features of the GP regression are similar across different choices of the time window *k* (embedding dimension). (*b*) Different values of T* for a fixed embedding dimension (time window) have a minimal effect in the backcasted result. Note that after T*, the observed values fall within the CI around the predictions (which is not depicted).

We demonstrate the effect of changing the time window on the BC result in figure 7*a*, whereas the effect of varying the T* is shown in figure 7*b*. In the latter, it can be seen that variations of T* do not change the qualitative picture described earlier. In the former, we can see that *k* = 13 may lead to potential overfitting, while already *k* = 10 yields an adequate qualitative representation of the number of cases.

**Figure 8.**
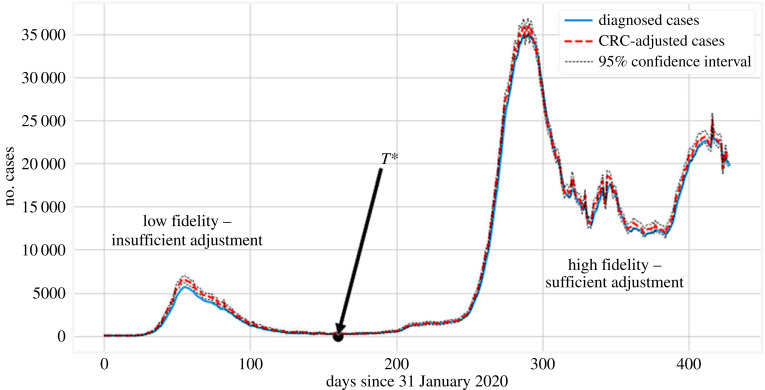
Demonstration of the CRC approach [21] to estimate uncertainty in the diagnosed cases. The CRC adjustment (presented in §2.3) to the number of cases is insufficient when the estimated number of cases is not realistic, which can be seen in comparison with the backcasted estimate of figure 5*b*. However, they may be considered a reasonable estimate of the true number of missed cases later on (${\hat{c}}_{t}$ for *t* ≥ T*).

**Figure 9.**
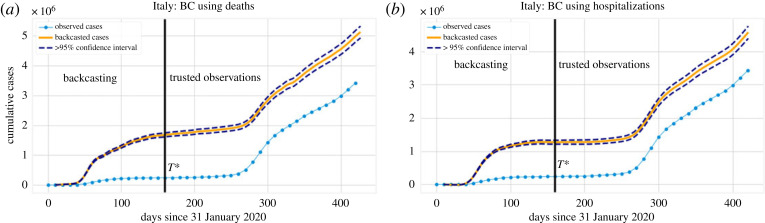
BC of the cumulative number of diagnosed cases for Italy using the CRC case estimate and uncertainty after T* = 160. Here, T* = 160, *k* = 10. (*a*) uses the data for deaths to perform the backcasting, while (*b*) uses that for hospitalizations. While the results are not identical, they are reasonably consistent.

The uncertainty estimate associated with the GP implementation is not necessarily accurate, since the obtained uncertainty intervals can be tuned using the kernel parameters. To avoid additional computation, we circumvent this issue using the same T* = 160 and time window *k* = 10, but apply the CRC method to adjust cases and their uncertainty after T* (as described in §2.3). The associated backcasted results are presented in figure 8. We note that in the low-fidelity region (first wave), the CRC re-adjustment does not alter significantly the number of cases. Moreover, the cumulative number of cases based on the CRC-adjusted BC is shown in figure 9*a*. Figure 9*b*, instead, shows BC results using the hospitalization time series in place of the fatalities time series as the predictor for cases. We note similar behaviour to figure 6, but with a much narrower uncertainty envelope. It is important to realize that the CRC adjustment of the cases can only be relied upon for *t* ≥ T* and not where the estimated number of cases is unrealistic.

### 3.2. Comparative results

We extend our considerations to Spain, Sweden (where official policies related to COVID-19 were notably very different from those in other EU countries), Germany, the United Kingdom, France and South Korea, as well as Brazil, with the last two representing rather extremely opposite-end examples of mitigation approaches towards the pandemic, as discussed later. These countries (with the exception of Brazil) fit the time series profile described in §2.2.1: they have a first peak, followed by a period over the summer of 2020 where reported cases ebbed and then a subsequent second peak during the autumn months of 2020. The particularity of the COVID-19 dynamics in Brazil, and its difference from that in India and the United States, was also noted in [26]. We speculate that the absence of the well-defined separation between the first and second waves, observed in the other countries studied herein, may be due to the saturation effect in hospital beds and intensive care units (ICUs).

Among these countries, Sweden has the distinction of not ordering a nationwide lockdown during the first wave of the pandemic, as the other European countries considered in this work did [46]. Instead, among the mandated measures adopted were self-isolation, social distancing, banning of public events and partial school closures. South Korea adopted a proactive approach and started developing testing capabilities almost two weeks before the first case in the country [47], established contact tracing protocols as early as mid-February of 2020, enforced social distancing from 22 March to 15 April, demanded tests for all incoming travellers and quarantine for travellers from selected countries and finally, redistributed resources at hospitals and emphasized the use of personal protective equipment by healthcare workers.

In sharp contrast, the lack of coordination at the federal level and delays in the implementation of mitigation measures in Brazil have been well documented [48,49]. Testing capacity in the country was very limited, with test kits being available for the first time in March 2020, whereas the first phase of the epidemic in Brazil started in the middle of February 2020. In addition, there were regions with extremely low ICU bed capacity, such as Amazonas, where the capacity was 11 beds for 100 000 people. As a result, Manaus, the capital city of Amazonas, was one of the regions that was affected particularly intensely. For instance, fatalities per 100 000 people due to COVID-19 in this region exceeded by a factor of two fatalities in the country overall.

**Figure 10.**
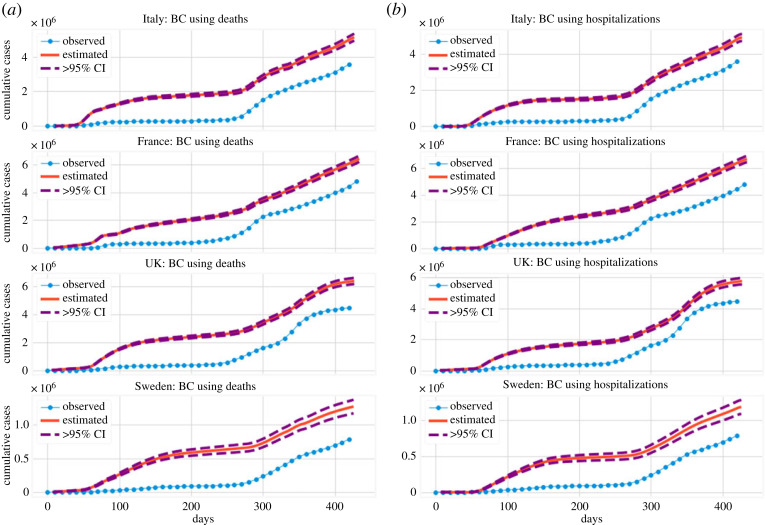
Backcasting of the cumulative number of diagnosed cases using deaths (*a*) and hospitalizations (*b*) as predictors. For all of the countries, we use T* = 160 and the corresponding embedding dimension estimated in table 1, as well a delay time of 1 day.

All country time series (except that of Brazil, as previously mentioned) satisfy the structure of §2.2.1 (see, also, figure 1*a*,*b*), and this structure extends to the corresponding hospitalization data, when available. In the results shown in figure 10, we use both *D* and *H* (hospitalizations, defined analogously to *D*, equation (2.1)) as predictors, with the uncertainty estimated using the CRC process [21]. The embedding dimension *k* was again estimated using the FNN algorithm, with the results presented in table 1. We find that the predicted, cumulative number of cases in the four countries illustrated in figure 10 and table 2 was larger by a factor typically between 1.3 and 2 when evaluated at the end time of our dataset (4 April 2021). However, the same factors vary greatly when computed at time T*, at which point the estimate is purely determined through the backcasted result.

**Figure 11.**
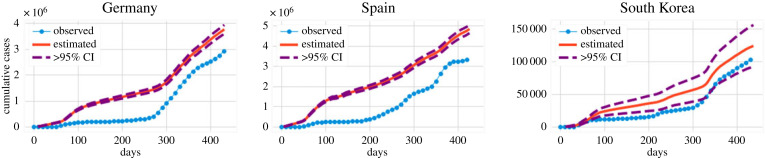
Backcasting results of the cumulative number of diagnosed cases for countries where daily hospitalization records are not (or are partially) available. For all the countries, we use T* = 160 and a window, embedding dimension, of 15 days, and the same kernel for the Gaussian process (Matérn kernel).

For Germany, Spain, South Korea and Brazil, daily hospital occupancy is not readily available in our dataset, and we only include the prediction based on the corresponding fatalities. Figure 11 presents the backcasted cumulative number of cases (in analogy to figure 10*a*), whereas table 3 presents the results of our calculations at *t* = 400 and compares them with the reported number of cases (analogously with table 2).

Two other studies performed backcasting of cases [21,46]. According to [21], which used a CRC method, the ratio of total to reported cases for Italy, Germany, Spain and the UK were 2.23, 2.30, 2.21 and 2.37, respectively. Instead, according to the results shown in tables 2 and 3, we find that the ratio of backcasted cumulative number of cases to the reported number is 1.43 (Italy), 1.85 (Germany), 2.00 (Spain) and 1.47 (United Kingdom). These numbers are smaller than those reported in [21], but they are consistent with the overall observation that the number of reported cases during the first epidemic wave is significantly smaller than the actual number of infected individuals. In [46], backcasting was performed from observed deaths based on a Bayesian mechanistic model; they found that until 4 May 2020, the percentage of population infected in Italy, France, the UK, Germany, Sweden and Spain was 4.6%, 3.4%, 5.1%, 0.85%, 3.7% and 5.5%, respectively. These far exceed the reported infections in each of these countries. In comparison, our results (in the same country order) are 2.8%, 2.8%, 3.3%, 1.2%, 5.9% and 4.5%. We note that these results are in the same order of magnitude as those of [46], although there are differences due to the detailed assumptions of each setting.

**Figure 12.**
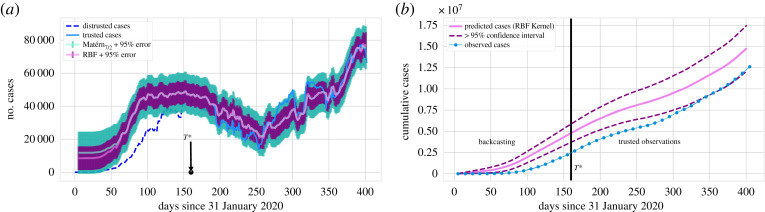
Backcasting results for Brazil with a time window of 5 days. (*a*) Results for two different choices of kernel (Matérn and RBF) for the GP regression. After time T* = 160, the predicted cases (pink solid line) are close to the observed cases (blue solid line, filled circles) since the model has been trained at those points. (*b*) One of the results (RBF) for the sake of clarity.

We also include the backcasting projection for Brazil: the number of daily cases is shown in figure 12*a*, whereas the cumulative number of cases is presented in figure 12*b*. There, the data do not satisfy the distinct wave structure of the previous examples. Irrespective of that, we found similar features as in the previously reported examples. In particular, we found that the diagnosed cases for $t\leq T^{⋆}$ were under-reported, although by a substantially smaller fraction than in other countries. Specifically, the proposed backcasting method predicts a higher and earlier peak in cases than observed, but it notably fails when trying to extrapolate outside of the range of the given data: early predictions, when cases and deaths due to COVID-19 are near zero, should yield a similar result. The left tail should therefore be considered inaccurate. We only include this example to demonstrate why the backcasting result may not be trustworthy if the structure of the data is not consistent with §2.2.1, even if it seems plausible.

## 4. Discussion and conclusion

We developed a computational framework that uses as input the case and fatality incidence in the second wave of the COVID-19 epidemic in various regions to evaluate the magnitude of case incidence of the first wave, assuming the incidence of fatalities is accurate. The framework is based on algorithms that leverage the Takens–Whitney delay embedding theorems [31,32,50], use the method of FNNs [39] to estimate the embedding dimension and employ Gaussian processes (GP) to perform nonlinear regression (as well as to quantify the resulting uncertainty). Uncertainty is also quantified using the CRC approach of [21].

The method requires a minimal number of external parameters besides those hyperparameters internal to the GP algorithm. These parameters include the embedding dimension *k*, the parameter T* and the time delay *τ*, which for the 7-day averaged time series was taken to be 1 day. The parameter *k* is the number of lagged observations required to construct the manifold $M_{D}$ diffeomorphic to M. The time parameter T* separates (somewhat artificially) the data into a segment past T*, after which the data are considered to be accurate (typically a low incidence time during the summer). Assuming the accuracy of fatalities (or hospitalizations), the aim is to backcast the incidences during the first wave, i.e. during the early stages of the pandemic, based on the trends between cases and fatalities. The relatively small number of parameters and hyperparameters sets the current methodology apart from other ordinary or partial differential equation (respectively, ODE or PDE) approaches, for which a significant effort is required to resolve parameter identifiability issues [22,51].

The framework was used with data from various European countries, which adopted disparate mitigation strategies, as well as with data from South Korea and Brazil, which also followed antipodal approaches to curtail the burden of the pandemic. Among the findings of this study is that in the six European countries studied (Italy, France, the United Kingdom, Sweden, Germany and Spain), the first wave was, as widely suspected, considerably larger than reported. Specifically, at the end of the period considered, the ratio of predicted (backcasted) cumulative number of infections to those reported was found to be 1.43 (Italy), 1.39 (France), 1.47 (UK), 1.69 (Sweden), 1.33 (Germany) and 1.57 (Spain), i.e. the predicted number of cases was consistently greater by over 30% than the reported number. In South Korea, however, where the epidemic was controlled, the discrepancy between predicted and observed cases is substantially smaller; we found the ratio of predicted to reported to be 1.22, a discrepancy of 18%. This is a case example, as was explained in the text, where a significantly different (and far more proactive) mitigation strategy was brought to bear, which is apparently reflected in our results.

There are numerous technical issues and further possibilities to consider along this line of efforts. Backcasting can be implemented between other time series, e.g. between deaths and hospitalizations. A relevant time series that has received considerable attention is that of ‘excess deaths’. This has been a traditional way to gauge the uncertainty around the death toll of exceptional events, such as the current pandemic [25] or extreme heat waves [52]. Excess deaths measure the deviation of the observed death toll from the expected death toll during a period of time, where the expected death toll is often a function (if not the direct average) of the death toll of the previous few years. While this is used to measure the overall impact of the pandemic, it takes into account increases or decreases in mortality due to other causes as well as rendering it at times not a reliable measure of the true deaths attributable to an exceptional event (such as, in this case, COVID-19). Because of the disruptive nature of the pandemic, it is almost certain that the excess deaths seen are not all attributed to the virus itself: e.g. the diversion of most medical resources to the care for COVID-19 patients decreased the availability of medical resources for the care of patients with other chronic illnesses or with non-COVID-19 acute symptoms. There are also countries that have not seen a rise in excess deaths despite confirmed deaths due to the coronavirus [53]. Australia and New Zealand recorded lower mortality than in recent years, which is attributed to social distancing measures that decreased mortality for reasons other than COVID-19, e.g. due to low incidence of seasonal flu. Uruguay and Norway, also reported negative excess deaths. In addition, in their analysis of the first and second waves, [37] found negative excess mortality data for four European counties (Denmark, Hungary, Lithuania and Norway) providing further doubts on the reliability of excess deaths to estimate COVID-19 deaths.

An important limitation of our approach, and the algorithm that infers earlier from later data, is associated with the accuracy of the reported case and death time series, and how that accuracy varies with the evolution of the pandemic. Numerous works, see, e.g. [26,28,37], questioned the improvement of the quality of reported data during the pandemic. They argued that data reporting depends on country-specific monitoring protocols that evolved during the pandemic, on possibly relaxed measures and on individual behavioural changes. While improved accuracy with time may not be guaranteed, the number of reported cases in the countries we studied increased considerably during the second wave. We, thus, consider that the number of reported cases during the second wave reflects the epidemiological state of the epidemic in those countries more accurately.

Trying to isolate true COVID-19 fatalities missed in the official numbers is a daunting (if not impossible) task, and that is acknowledged in the relevant literature as well [23]. Statistically (and if we disregard the concerns of the earlier paragraph), we can perform regression between the observed and excess deaths to find what percentage of deaths was missed. This is a process that must be done individually for each country, and its accuracy depends on the availability and quality of datasets, accumulated over several years.

An alternative method to quantify the magnitude of the true number of cases, which has been put in practice in various regions, is to monitor the viral load carried in sewer waste [54,55]. It would be useful to examine the relevant correlations in subsequent waves vs. the measurements during the first wave. In a scenario where significant testing takes place (as, e.g. is the case in South Korea among our selected examples) or was subsequently developed, perhaps one can use the correlation between fatalities, symptomatic and asymptomatic cases, to infer the fractions of infections that stem from symptomatic vs. asymptomatic cases. This is an interesting aspect of such epidemiological models for which presently there is considerable uncertainty with very different asymptomatic fractions being reported in different studies [15].

## Data Availability

Data and relevant code for this research work are stored in GitLab: https://gitlab.com/peacogr/backcasting and have been archived within the Zenodo repository: https://doi.org/10.5281/zenodo.7235554 [56]. Our COVID-19 data source is the publicly available Our-World-In-Data repository on Github, which compiles different types of data from multiple primary sources. Links and the specific version of the dataset used in our results are provided at the project page linked earlier. The data are provided in electronic supplementary material [57].

## Footnotes

1
https://github.com/manu-mannattil/nolitsa

2
https://scikit-learn.org/stable/modules/gaussian_process.html

3
https://CRAN.R-project.org/package=R0
